# Retraction Note: Decompressive craniectomy without durotomy for traumatic coma and uncontrollable intracranial hypertension

**DOI:** 10.1186/s13032-016-0032-y

**Published:** 2016-01-22

**Authors:** Daniel James Miller, Darlene Angela Lobel

**Affiliations:** Department of Surgery, Division of Neurosurgery, University of California, San Francisco-Fresno, 2335 East Kashian Lane, Suite 301, Fresno, California 93701 USA; Department of Neurosurgery Center for Neurological Restoration, Neurologic Institute, Cleveland Clinic, 9500 Euclid Avenue, S31, Cleveland, Ohio USA

The Publisher has retracted this article [1] because it appears that the authors did not obtain the necessary consent to publish their case details from the patients described. The article is no longer available online in order to protect the patients’ privacy. Darlene Lobel has agreed to retraction, Daniel Miller could not be reached by the journal for comment on the retraction.
